# Underfueled and overloaded: association between low energy availability symptoms and current self-reported injury status in climbers

**DOI:** 10.3389/fspor.2026.1886526

**Published:** 2026-08-17

**Authors:** Gudmund Grønhaug, Nicholas Slagel, Atle Hole Saeterbakken

**Affiliations:** 1 Department of Sport, Food and Natural Sciences, Western Norway University of Applied Sciences Faculty of Teacher Education and Sport, Sogndal, Norway; 2 Department of Kinesiology, Nutrition and Dietetics, University of Northern Colorado, Greeley, CO, United States

**Keywords:** climbing, eating disorders, energy availability, nutrition, REDs, sports injuries

## Abstract

**Objective:**

The objective of this study was to investigate whether low energy availability (LEA) symptom burden is associated with current self-reported climbing-related injury status.

**Methods:**

A cross-sectional, web-based survey was conducted to assess current self-reported climbing-related injury status, LEA symptoms, eating pathology, and psychological distress using validated and previously published instruments. Multivariable logistic regression models were applied to estimate odds ratios (ORs) for reporting a current injury, adjusting for age, sex, level of performance, training frequency, and climbing frequency. Sensitivity and interaction analyses were performed to evaluate psychosocial and climbing-context moderators.

**Results:**

A total of 417 climbers participated (mean age 33.1 ± 9.9 years; 56.2% female). Higher-ability climbers showed greater odds of reporting a current climbing-related injury than lower-ability climbers (OR 2.10, 95% CI 1.33–3.30). LEA symptom burden was independently associated with current self-reported injury status (OR 1.38, 95% CI 1.08–1.76). The association remained significant after adjusting for anxiety and climbing discipline but was attenuated after adjusting for eating pathology and depressive symptoms. Eating pathology, depression, and anxiety were not independently associated with injury. Exploratory interaction analyses suggested that the association between LEA symptom burden and current self-reported injury status may be stronger among climbers scoring higher on eating pathology and higher climbing ability; however, these interactions were not statistically significant and should therefore be interpreted cautiously. Gastrointestinal symptoms were the LEA domain most consistently associated with self-reported current injury status.

**Conclusion:**

Greater LEA symptom burden was independently associated with current self-reported climbing-related injury status after adjusting for demographic characteristics and climbing exposure. Gastrointestinal symptoms demonstrated the most consistent association with current self-reported injury status. Higher climbing ability and LEA symptom burden were also associated with greater odds of reporting a current injury. However, there is a need for prospective longitudinal studies to clarify the temporal and potentially causal relationships among energy availability, psychological health, training load, and injury outcomes.

## Introduction

Climbing is both an organized elite competition sport and an unorganized sport engaged in by recreational, semiprofessional, and professional athletes. The popularity of climbing has increased rapidly over the past two decades, driven by the proliferation of new climbing centers and facilities, inclusion in the Olympic Games, and widespread coverage in social and mainstream media. The growth has been notable across all disciplines of climbing, such as bouldering, sport climbing, and traditional climbing (1–3). Many recreational climbers train in a manner similar to competitive athletes, with high training volumes, structured periodization, and performance expectations creating physiological demands comparable to high-level athletes (4–6). As participation and training volume continue to increase, understanding the factors associated with current injury and athlete health has become increasingly important for clinicians, coaches, and climbers.

Current climbing-related injury is likely influenced by multiple interacting factors, including mechanical loading, training exposure, recovery, nutrition, and psychological health. Mechanical loading and climbing ability have consistently been associated with climbing-related injury, with higher-performance climbers generally experiencing greater cumulative loading because of increased training volume and intensity (7–9). These factors provide important context when interpreting injury patterns but do not fully explain why some climbers report persistent injury while others with similar training characteristics remain uninjured.

Low energy availability (LEA) can impair endocrine, metabolic, and neuromuscular systems and has emerged as a potential contributor to impaired physiological functioning and injury in athletes (10–14). Rather than representing a single disease process, RElative energy Deficiency (REDs) describe a constellation of physiological adaptations that may occur when energy availability is chronically inadequate.

Emerging evidence suggests that climbers may be particularly susceptible to LEA-related symptoms because climbing performance often rewards a high strength-to-mass ratio, and some climbers intentionally pursue lower body mass to improve performance (15–18). Previous studies have reported inadequate energy intake, restrictive eating behaviors, weight-control practices, and symptoms consistent with LEA among climbing populations (17–19). However, relatively little is known about how LEA-related symptom burden relates to the presence of current climbing-related injury in this population.

Research using LEA screening tools, such as the Low Energy Availability in Females Questionnaire (LEAF-Q) and the Low Energy Availability in Males Questionnaire (LEAM-Q), has demonstrated possible associations with injury, impaired recovery, and altered physiological function in multiple athlete groups (19–22). Importantly, most of these studies have been observational, and the presence of LEA symptoms should not be interpreted as evidence of causal mechanisms for injury. Instead, LEA symptoms may coexist with injury because both are influenced by common underlying factors such as training load, recovery practices, nutritional behaviors, and overall athlete health. In a state of LEA persistent fatigue, reduced recovery capacity, sleep disturbance, and thermoregulatory changes may compromise tissue health and neuromuscular performance (23–25). Nevertheless, research exploring LEA within climbing populations remains limited, with only a handful of studies examining whether symptom burden is linked to current self-reported injury status (26–28).

Disordered eating may contribute to the development of LEA. However, it should not be considered a direct physiological mechanism for injury independent of energy availability (10, 15, 29). Although restrictive eating behaviors may contribute to inadequate energy availability, many athletes with LEA do not meet criteria for an eating disorder, and conversely, eating pathology may coexist with injury through mechanisms other than energy deficiency. Although some studies have linked weight-control pressure to some subgroups in climbing (26, 27), no studies have examined associations between disordered eating and current self-reported injury status in climbers. Similarly, although gender differences in REDs manifestations are well documented (10–21, 29), sex differences in the association between LEA symptoms and current self-reported injury status are not known. Thus, whether eating pathology contributes independently to current injury status or primarily reflects broader LEA-related symptom burden remains unclear.

Psychosocial factors such as stress, anxiety, and depressive symptoms have been associated with sport injury risk. Meta-analytic evidence has linked psychosocial variables to injury occurrence, with prospective evidence showing that preseason anxiety and depressive symptoms predict injury incidence in collegiate athletes (30–32). In climbing-specific samples, clinically meaningful depression, anxiety, and stress symptoms co-occur with high rates of overuse problems and poor sleep quality (33). Together with REDs frameworks that recognize psychological disturbances and sleep disruption as indicators and consequences of problematic LEA (11–13), these findings support the evaluation of depression and anxiety as exploratory covariates and moderators. This may help clarify whether LEA symptom burden (including sleep and fatigue domains), eating pathology, and self-reported current injury are independently associated or linked through overlapping psychosocial pathways. The aim of the present study was to investigate whether LEA symptom burden is associated with self-reported current injury status among sport climbers after adjusting for age, sex, climbing ability, and training exposure. In addition, we sought to explore whether climbing discipline, eating pathology, and psychological distress attenuate or modify these associations through planned sensitivity and interaction analyses.

## Methods

The PANIC (Pain, Anxiety, and Injury in Climbing) study is a broader cross-sectional survey project designed to examine injury, pain, psychological factors, nutritional factors, and climbing-related behaviors in recreational and competitive climbers. The present analysis used selected PANIC variables relevant to self-reported current injury status, LEA symptom burden, eating pathology, psychological distress, climbing ability, and training exposure. Standardized instruments were employed for nutrition and eating behaviors, depression and anxiety, and LEA symptomology. Sociodemographic, climbing exposure, goal, and injury-related items were investigator-developed or adapted from prior climbing injury surveys. The questionnaire was published via Qualtrics as a web-based item-driven questionnaire. The questionnaire was open for respondents from December 2024 to end of March 2025. Participants were recruited using convenience sampling methods, including social media outreach, word-of-mouth referral, and flyers posted in local climbing gyms. Inclusion criteria were age ≥18 years and current participation in indoor and/or outdoor climbing. The study protocol was approved by the University of Northern Colorado Institutional Review Board (IRB #2409062358), and all participants provided informed consent prior to participation. The full survey is available in Supplementary Tables.

### Measures

#### Injury

Questions regarding injury, aim of training, and injury-related behavior were based on previously published surveys (34). For the present study, only one question from the questionnaire was used: “Do you have any current climbing related injury? Yes/no.” No medical verification was performed. The item used is one of several questions on injury and pain. The questionnaire had an instruction text defining the injury as chronic rather than acute. Time loss and treatment were also measured but not used in this study. Consequently, the outcome should be interpreted as self-reported current injury status rather than clinically verified injury.

#### Low energy availability

In the broader study, LEA symptom burden was assessed using the LEAM and the LEAF (35). For the present injury analyses, the primary LEA-related exposure was a revised symptom-burden composite of the LEAM questionnaire. Injury-related items were removed to avoid criterion contamination with the injury outcome. Sex-drive items 3–4 were excluded because they were male-specific and not appropriate for a cross-sex total; sex-drive items 1–2 were retained because they reflected general sex-drive symptoms that may apply across genders. The revised composite was used to quantify symptom burden and was not interpreted using validated LEAM diagnostic thresholds. Because the modified composite has not undergone independent validation, findings should be interpreted with appropriate caution. Exploratory domain analyses employed the revised LEAM symptom-burden composite domains which included dizziness/vision, gastrointestinal symptoms, thermoregulation, fatigue A (systemic fatigue), fatigue B (physical strain), sleep, recovery, energy/mood, and sex drive (items 1–2 only).

#### Eating disorders and disordered eating

Eating disorder symptom severity was assessed with the 12-item Eating Disorder Examination Questionnaire–Short (EDE-QS) (25), using the recommended screening threshold of ≥15 for elevated eating disorder risk (26).

#### Psychological distress

Psychological distress was assessed using the 21-item Depression Anxiety Stress Scales (DASS-21) (36), which captures symptom severity across depression, anxiety, and stress domains over the prior week. Item responses were recoded from 1 to 4 to 0–3, subscale totals (7 items each) were summed and multiplied by 2 to align with standard DASS-21 severity cut-points (0–42 metric). DASS-21 subscales were used descriptively to characterize distress burden in the sample and to support inferential analyses.

### Analysis methods

Data were screened for missingness and distributional assumptions. Continuous variables are reported as mean ± SD and categorical variables as *n* (%). Sex and performance-level group comparisons used independent-samples *t*-tests for continuous outcomes and *χ*^2^ tests for categorical outcomes.

The primary outcome was self-reported current injury status (yes/no). Primary inferential analyses used multivariable logistic regression to estimate odds ratios (ORs) and 95% CIs for current injury status as a function of LEA symptom burden, operationalized as a revised LEAM-derived symptom-burden composite. Primary adjusted models included *a priori* covariates reflecting demographic and exposure-related injury determinants: age, self-reported gender, climbing ability, and training exposure (training frequency and climbing frequency). Gender was self-reported and coded as male, female, or other gender. In regression models, male was the reference category, with female and other gender entered as indicator variables. Because the number of participants reporting other gender was small, gender-specific interpretations are limited. The grouping of the level of performance in this study was first used in Grønhaug and Norberg (4). For analysis, ability categories were collapsed into lower ability (beginner–intermediate) vs. higher ability (advanced–elite) to provide a parsimonious proxy of climbing exposure/training load and to ensure adequate cell sizes for regression models. Climbing frequency represented time spent climbing, whereas training frequency represented non-climbing training activities such as strength and conditioning. Both variables were included to capture potentially distinct sources of physical load.

Continuous psychosocial predictors (revised LEAM-derived symptom-burden composite total, EDE-QS total, and DASS-21 subscales) were standardized (*z*-scores) for interpretability and comparability of effect estimates (OR per 1 SD increase). Multicollinearity was assessed using variance inflation factors and tolerance values from linear regression diagnostics fitted to the same predictor sets as the logistic regression models. Variance inflation factors were low in the primary and planned sensitivity models (maximum VIF=1.81; minimum tolerance=0.55), indicating no evidence of problematic multicollinearity. Exploratory interaction models had higher, but still moderate, variance inflation factors because of product terms, and these models were interpreted cautiously.

Sensitivity analyses were conducted to evaluate whether the LEA symptom burden–self-reported injury association remained robust after adding psychosocial constructs that may be related to both LEA symptom burden and injury status. Eating pathology (EDE-QS) (11, 12) was examined in sensitivity models. Psychological distress was assessed with the DASS-21 (36) and examined in separate sensitivity models by adding standardized depression or anxiety scores to the primary injury model to evaluate attenuation of the LEA symptom burden–self-reported injury association. Depression and anxiety were modeled separately to avoid overadjustment and reduce collinearity among related psychological distress constructs.

Effect moderation analyses were conducted to test whether the association between LEA symptom burden and injury differed across psychosocial factors or climbing disciplines. Interaction models tested whether eating pathology modified the association between LEA symptom burden and current injury status, whether eating pathology effects differed by sex, and whether psychological distress modified the LEA–injury relationship. Given discipline-specific loading patterns in climbing, additional contextual models were restricted to participants identifying bouldering or route/sport climbing as their primary discipline. These models examined bouldering discipline and ability as covariates and tested LEA × bouldering, LEA × ability, and bouldering × ability interaction terms. Analyses involving psychological distress variables, interaction terms, and domain-specific LEA components were considered exploratory and were interpreted accordingly.

Complete-case data for the primary current injury model included 390 participants, of whom 100 reported a current climbing-related injury and 290 did not. The EDE-QS, depression, anxiety, and psychosocial interaction models used the same analytic sample. Contextual models restricted to bouldering and route/sport climbers included 378 participants, of whom 97 reported a current injury and 281 did not. The primary and sensitivity models generally exceeded 10 injury events per predictor, whereas the contextual interaction and all-domain exploratory models had fewer events per predictor and were therefore interpreted cautiously.

Descriptive tables include all participants who met inclusion criteria and completed the survey, with valid *n* reported for each variable. No *a priori* power calculation was conducted for this exploratory cross-sectional analysis. For the modified LEAM-derived symptom-burden descriptive analyses, complete data for the composite were available for *n* = 407. Regression analyses used complete-case data for the outcome, modified LEAM-derived symptom-burden composite, and required covariates; therefore, analytic *n* varied by model and is reported for the primary and contextual model families. All tests were two-sided with *α *= 0.05. Because secondary and exploratory analyses were hypothesis-generating, no formal adjustment for multiple comparisons was applied. Analyses were conducted using IBM SPSS Statistics (version 29; IBM Corp, Armonk, NY, USA).

## Results

### Sample characteristics

A total of 417 adult sport climbers completed the survey. Participants were 33.1 ± 9.9 years old, 56.2% were female, and most were from the United States or Europe. Most participants reported bouldering or route/sport climbing as their primary discipline, and 14.4% reported competition climbing. Overall, 62.3% were classified as lower ability and 37.7% as higher ability (Table 1).

**Table 1:** Participant, climbing, and training characteristics.

Characteristic	Value
Age (years)	33.1 ± 9.9 (*n* = 417; range, 18–78)
Gender (*n* = 411)	
Female	231 (56.2)
Male	164 (39.9)
Another gender	16 (3.9)
BMI (kg/m^2^)	24.3 ± 3.2 (*n* = 197; range, 18.13–30.0)
Geographic region (*n* = 395)	
United States	198 (50.1)
Europe	153 (38.7)
North America (excluding US)	21 (5.3)
Oceania	18 (4.6)
Asia	2 (0.5)
South America	3 (0.8)
Primary climbing discipline (*n* = 417)	
Bouldering	223 (53.5)
Route/lead climbing	177 (42.4)
Traditional climbing	14 (3.4)
Other	3 (0.7)
Climbing ability (*n* = 414)	
Lower ability (beginner–intermediate)	258 (62.3)
Higher ability (advanced–elite)	156 (37.7)
Competition climbing	60 (14.4)
Average time climbing (%)	
Indoor	75.44 ± 25.96
Outdoor	24.24 ± 25.64
Primary climbing goal (*n* = 417)	
Climb harder	270 (64.7)
Stay at the same level	33 (7.9)
General fitness	51 (12.2)
Improved general fitness	63 (15.1)
Training frequency, h/week (*n* = 417)	
Low (<3)	220 (52.8)
Moderate (4–7)	128 (30.7)
High (>8)	69 (16.5)
Climbing frequency, h/week (*n* = 417)	
Low (<3)	96 (23.0)
Moderate (4–7)	222 (53.2)
High (>8)	99 (23.7)

Percentages may not sum to 100% due to rounding and/or missingness. BMI was cleaned by restricting to 17–30 kg/m^2^ due to implausible self-entered values.

### Injury

Current injury prevalence was reported by 26.9% of participants. Injury prevalence did not differ by sex or climbing type.

However, current injury prevalence was more common among higher-ability climbers than lower-ability climbers, with approximately twice the odds of reporting a current injury (Table 2).

**Table 2:** Self-reported current injury prevalence by discipline and ability in the bouldering and route/sport subset.

Group	No injury, *n* (%)	Current injury, *n* (%)	Chi-square (df)	*p*	Odds ratio	95% CI
Route/sport	136 (76.8)	41 (23.2)				
Bouldering	159 (71.3)	64 (28.7)	1.56 (1)	0.211	1.34	0.85, 2.10
Lower ability (Beg–Int)	196 (79.4)	51 (20.6)				
Higher ability (Adv.–Elite)	99 (64.7)	54 (35.3)	10.47 (1)	0.001	2.10	1.33, 3.30

Percentages are within discipline and ability groups. The discipline comparison is restricted to participants whose primary discipline was bouldering or route/sport climbing; traditional and other climbing categories were not included because of small cell sizes. Ability comparisons include participants with complete current injury and ability data. Odds ratios compare bouldering with route/sport climbing and higher ability with lower ability.

### Low energy availability

LEA symptom burden did not differ meaningfully by climbing discipline or ability group.

In unadjusted comparisons of LEA domains, participants with a current injury reported higher total LEAM total scores and elevated scores for several symptom domains such as dizziness, gastrointestinal symptoms, thermoregulation, systemic fatigue, sleep, and recovery. Energy/mood showed a borderline difference, sex drive did not differ, and physical strain scores were lower among injured climbers (Supplementary Table S1).

### Eating disorder and disordered eating

The mean EDE-QS total score was 6.13 ± 5.99 (range, 0–31), with 9.4% of participants scoring above the EDE-QS screening threshold. Females climbers had higher mean EDE-QS scores than males (6.90 vs. 4.80; *p* < 0.001) and a higher proportion above the screening threshold (11.3% vs. 5.5%; *p* < 0.05).

### Psychological distress

Overall, mean stress symptoms were higher than depression and anxiety. Approximately one in five participants screened in the moderate or higher range for depression (21.0%) and anxiety (21.3%), and 18.3% screened moderate or higher for stress, indicating that clinically meaningful distress was common in this climbing cohort. DASS-21 descriptive statistics and internal consistency estimates are provided in Supplementary Table S2).

### Primary and sensitivity models

The primary complete-case model included 390 participants, of whom 100 reported a current climbing-related injury and 290 did not. In the primary adjusted model, LEA symptom-burden total and higher climbing ability were both independently associated with higher odds of injury. Each 1-SD increase in LEA symptom burden was associated with higher odds of current injury, and higher-ability climbers had more than twice the odds of injury compared with lower-ability climbers. Age, sex, training frequency, and climbing frequency were not independently associated with current injury.

Sensitivity analyses showed a similar pattern. The LEA symptom burden–self-reported injury association remained significant after adjusting for anxiety and in the bouldering-context model but was attenuated after adjusting for EDE-QS and depression. EDE-QS, depression, anxiety, and bouldering were not independently associated with current injury, whereas higher ability remained consistently associated across models (Table 3).

**Table 3:** Primary and psychosocial sensitivity models of LEA symptom burden and injury.

Model	LEAM effect OR (95% CI); *p*	EDE-QS *z* OR (95% CI); *p*	Depression *z* OR (95% CI); *p*	Anxiety *z* OR (95% CI); *p*	Bouldering OR (95% CI); *p*	Higher ability OR (95% CI); *p*
Primary	1.377 (1.079–1.758); 0.010	—	—	—	—	2.308 (1.347–3.955); 0.002
+EDE-QS	1.292 (0.998–1.672); 0.052	1.216 (0.942–1.570); 0.132	—	—	—	2.390 (1.389–4.113); 0.002
+Depression	1.196 (0.897–1.596); 0.222	—	1.283 (0.983–1.674); 0.067	—	—	2.326 (1.355–3.991); 0.002
+Anxiety	1.315 (1.012–1.709); 0.040	—	—	1.127 (0.874–1.452); 0.356	—	2.338 (1.362–4.013); 0.002
+Bouldering	1.385 (1.079–1.777); 0.010	—	—	—	1.057 (0.625–1.787); 0.836	2.247 (1.273–3.966); 0.005

Primary and psychosocial sensitivity models were adjusted for age, sex, climbing ability, training frequency, and climbing frequency. The modified LEAM-derived symptom-burden composite excludes injury items and sex drive items 3–4 while retaining sex drive items 1–2. Continuous predictors were standardized (*z*-scores), so ORs reflect a 1 SD increase. Primary, EDE-QS, depression, anxiety, and psychosocial interaction models used *n* = 390; the bouldering model used *n* = 378.

### Moderation analyses

In exploratory interaction models, the LEA symptom burden–self-reported injury association appeared stronger at higher EDE-QS scores. Sex, depression, and anxiety did not modify the LEA symptom burden–self-reported injury association. Higher climbing ability remained independently associated with injury across all psychosocial interaction models. However, there was no evidence that the LEA symptom burden–self-reported injury association differed by bouldering preference or ability, and the bouldering × ability interaction was also not significant (Supplementary Table S3).

### Domain-specific LEA symptom associations with current self-reported injury status

Exploratory domain models suggested that several LEAM domains were associated with current injury when examined individually. In multivariable domain models, most associations were attenuated, indicating overlap across symptom domains. GI symptoms remained positively associated with injury in both the full and parsimonious domain models, whereas fatigue B showed an inverse association in the full domain model. Higher ability remained associated with injury in both domain-adjusted models (Supplementary Tables S4–S6).

## Discussion

The present study identified an association between greater LEA symptom burden and current self-reported climbing-related injury status among recreational climbers. In addition, higher climbing ability was consistently associated with greater odds of reporting a current injury. These associations remained evident after adjusting for demographic characteristics and training exposure, although the association between LEA symptom burden and current injury status was attenuated after accounting for eating pathology and depressive symptoms. These findings indicate that LEA symptom burden is associated with current self-reported injury status; however, the cross-sectional design precludes conclusions regarding causality or underlying mechanisms. The findings are consistent with previous literature reporting associations between LEA symptoms, REDs related physiological disturbances impaired recovery, and injury-related outcomes (10–16, 30).

However, these findings should not be interpreted as demonstrating that LEA independently causes injury. Rather, LEA symptom burden may represent one component of a broader physiological and behavioral profile characterized by altered recovery, nutritional challenges, psychological distress, and cumulative training demands. Injury itself may also contribute to many of these symptoms through reduced activity, altered eating behaviors, pain, sleep disturbance, or psychological responses to injury. Consequently, the observed relationships are likely to be complex and potentially bidirectional.

Sport-specific research indicates that LEA may affect multiple body systems, particularly the gastrointestinal tract (37–43). These studies demonstrate that higher levels of performance yield higher energy expenditure (44) and emphasize the need for qualified clinical assessment (13, 45, 46). Recent critiques further caution that REDs-like symptoms are generic and may reflect multiple interacting stressors, including training load, poor sleep, psychological distress, disordered eating, injury, illness, and other clinical conditions (47).

Climbing ability was found to be an independently associated with current self-reported injury, with higher-ability climbers showing more than double the odds of injury compared with lower-ability climbers. This finding is consistent with previous climbing research demonstrating that more advanced climbers generally accumulate greater mechanical loading through higher training volumes, climbing intensity, and more frequent exposure to strenuous movements (43, 44). Still, higher level of performance did not modify the LEA symptom burden–self-reported injury association, suggesting that LEA symptom burden is an additive rather than an interactive effect with higher level of performance. These findings are in line with previous research linking higher level of performance with higher mechanical demands and training load, and thereby a higher need for energy (1–3, 7, 8). A lower-level climber may report a similar rate of perceived exertion when climbing an easier route as a higher-level climber does on a more difficult route. However, when considering relative load and maximal capacity, the physiological and perceptual demands may be comparable despite differences in absolute difficulty.

The lack of association between climbing discipline and injury after adjusting, and the absence of LEA symptom burden×discipline interaction, indicates that the LEA-related symptom profile observed in this study was not specific to the mechanical demands of bouldering versus route/lead climbing. Although these disciplines differ in movement demands and loading patterns (29), broad discipline categories may not capture the exposure variables most relevant to injury, such as intensity relative to ability, recent changes in training load, rest, recovery, or climbing while symptomatic. Sex was also not associated with current injury and did not modify the LEA symptom burden–injury association. The present data suggest that LEA-related symptom burden may be relevant across male and female sport climbers (4).

Training and climbing frequency were not independently associated with current self-reported injury in the adjusted model, suggesting that broad frequency categories may not adequately capture the loading patterns most relevant to injury. Frequency alone does not distinguish between chronic exposure, recent load spikes, climbing intensity relative to ability, accumulated fatigue, rest days, or behavioral changes after injury such as reducing volume or climbing at lower intensity. Consequently, the absence of statistically significant associations should not be interpreted as evidence that training load is unrelated to injury; rather, more comprehensive prospective load monitoring is required to distinguish harmful load spikes from protective chronic exposure and clarify dose–response relationships between external load, internal load, and injury (27–30).

An interesting finding was that gastrointestinal symptoms were the LEA domain most consistently associated with current self-reported injury status across the adjusted analyses. Gastrointestinal disturbances are recognized manifestations of prolonged low energy availability and may reflect alterations in gastrointestinal function, nutritional practices, psychological stress, or combinations of these factors (37–39). The inverse fatigue B (physical strain) association should be interpreted cautiously. This domain contains a heterogeneous mix of musculoskeletal discomfort, perceived vulnerability, exhaustion, and a reverse-coded strength/progress item. Thus, the results may capture training status or recent loading/recovery context rather than a straightforward LEA symptom-burden construct, suggesting that some apparent domain-level associations reflect overlapping variance across symptoms rather than independent injury signals.

The interaction analyses suggest that the association between LEA symptom burden and current self-reported injury status may be stronger among climbers with higher levels of eating pathology scores. This pattern supports an interpretation of multifactorial etiology, rather than constituting an independent injury mechanism once energy status is considered. Restrictive eating behaviors, weight-control pressures, and body- or food-related concerns may increase the relevance of physiologic symptoms without necessarily acting as an independent injury mechanism once symptom burden is modeled. This interpretation is consistent with climbing-specific evidence documenting nutrition knowledge gaps, intentional weight-loss practices, and disordered eating risk in climbing populations (17–19). Restrictive eating behaviors may contribute to reduced energy availability, while depression may influence appetite, motivation, recovery behaviors, sleep quality, and training participation. Conversely, current injury may itself contribute to depressive symptoms and altered eating behaviors through pain, frustration, reduced participation, or changes in daily routine. Because all variables were measured simultaneously, the present analyses cannot determine the direction or sequence of these relationships. Future longitudinal studies should examine how these factors evolve over time and whether they independently or jointly influence injury occurrence and recovery.

Similarly, anxiety symptoms were not independently associated with current injury status after adjusting for potential confounders. Although psychological distress has been associated with injury outcomes in previous prospective studies (31, 32), in practice, distress may influence sleep, appetite, pain perception, training decisions, recovery behaviors, and willingness to seek care. The attenuation observed when depression was included suggests overlap between depressive symptoms and LEAM domains such as fatigue, sleep, and recovery, rather than a separate injury pathway. Because the study was cross-sectional, psychological distress may precede underfueling behaviors, emerge after injury, co-occur with training stress, or reflect shared symptoms captured by the LEAM composite. Recent climbing-specific work showing co-occurrence of mental health symptoms, poor sleep, and overuse problems supports assessing these domains together while avoiding causal claims from cross-sectional data (33).

From an applied perspective, the results of the present study reinforce the value of multidisciplinary, athlete-centered support. Participation in high-performance sport is associated with inherent injury exposure. Inadequate energy availability disrupts endocrine, metabolic, neuromuscular, and psychological functioning. However, given that the majority of our sample comprised intermediate-level climbers who do not compete at high levels, they may not have access to multidisciplinary physicians, sport dietitians, or structured athlete-monitoring systems for climbers (13, 46, 48, 49), A more feasible implication is the need for evidence-based, low-barrier education and referral tools that help climbers recognize persistent symptom clusters and understand when professional support may be warranted. These resources could include brief symptom awareness checklists, climber-facing education on fueling and recovery, coach- or gym-facing referral pathways, and examples of how training load, eating behaviors, recovery, and symptom persistence may interact.

The findings suggest that adequate energy intake may be an important component of athlete health and recovery. However, the present study cannot determine whether increasing energy intake would reduce injury occurrence among the respondents. Training plans should incorporate structured load progression, dedicated recovery strategies, and nutrition education around the energy demands of different periods of training. Where applicable, coaches and practitioners should promote a climate where athletes feel comfortable reporting symptoms and where conversations focus on performance-related functioning rather than body weight or leanness. These recommendations are consistent with REDs consensus guidance (11–13, 21–23, 27–30). A recent climbing-specific pilot suggests that sport-specific nutrition education can improve nutrition knowledge in youth competitive climbers, but evidence remains limited regarding effects on eating disorder risk, REDs symptoms, injury outcomes, or adult recreational populations (49). Climbers showing signs of long-lasting and otherwise unexplained fatigue, low motivation, poor recovery, psychological distress, sleep disturbance, thermoregulatory complaints, or plateauing in performance over longer periods should be evaluated for physiologic drivers and energy status as defined by the REDs framework.

### Strengths and weaknesses

The present study has several strengths. This study represents one of the largest investigations to date examining associations among LEA symptom burden, eating pathology, psychological distress, and current self-reported climbing-related injury status. The inclusion of multiple validated questionnaires allowed simultaneous evaluation of nutritional, psychological, and climbing-related characteristics while accounting for several important demographic and training-related covariates. Furthermore, the analyses included sensitivity models that explored the influence of eating pathology, psychological distress, climbing discipline, and training characteristics on the observed associations.

Several limitations should also be acknowledged. First, the cross-sectional design precludes conclusions regarding temporality or causality. It is therefore not possible to determine whether greater LEA symptom burden preceded current injury, resulted from injury, or reflected shared underlying determinants. Second, injury status was based on participant self-report and was not medically verified. Consequently, misclassification of injury type, diagnosis, severity, or chronicity cannot be excluded. Third, the modified LEAM-derived symptom-burden score has not undergone independent psychometric validation following removal of injury-related items and therefore should be interpreted cautiously. Fourth, although training and climbing frequency were included as covariates, these measures provide only limited information regarding cumulative mechanical loading, recent changes in workload, recovery practices, or training intensity. Fifth, complete-case analyses may have introduced selection bias if missing data were not completely random. Finally, the exploratory interaction and domain-specific analyses were not adjusted for multiple comparisons and should therefore be considered hypothesis-generating rather than confirmatory.

Taken together, these limitations indicate that the present findings should be interpreted as identifying associations among coexisting characteristics within climbers rather than establishing causal pathways or informing preventive interventions.

## Conclusion

Greater LEA symptom burden was independently associated with current self-reported climbing-related injury status after adjusting for demographic characteristics and climbing exposure. Gastrointestinal symptoms demonstrated the most consistent association with current self-reported injury status. Higher climbing ability and LEA symptom burden were also associated with greater odds of reporting a current injury. However, there is a need for prospective longitudinal research to clarify the temporal and potentially causal relationships among energy availability, psychological health, training load, and injury outcomes.

## Data Availability

The original contributions presented in the study are included in the article/Supplementary Material, further inquiries can be directed to the corresponding author/s.
