# Supplementary material for: Underfueled and overloaded: association between low energy availability symptoms and current self-reported injury status in climbers
Source: Front Sports Act Living. 2026 Aug 17;8:1886526. doi: 10.3389/fspor.2026.1886526 (PMC13526988; doi:10.3389/fspor.2026.1886526)
Supplement: Supplementary file 1 [file Table1.docx]

*Supplementary Tables*

**Supplementary Table S1. LEAM scores by current injury status**

| **LEAM measure** | **Total sample** | **Current injury: Yes** | **Current injury: No** | **p-value*** |
| --- | --- | --- | --- | --- |
| LEAM total | 51.99 ± 9.96 (n=407) | 54.28 ± 10.64 (n=108) | 51.16 ± 9.59 (n=299) | 0.005 |
| Dizziness/vision symptoms | 0.87 ± 1.21 (n=407) | 1.14 ± 1.41 (n=108) | 0.78 ± 1.12 (n=299) | 0.018 |
| GI symptoms | 7.59 ± 1.30 (n=407) | 7.92 ± 1.53 (n=108) | 7.48 ± 1.19 (n=299) | 0.008 |
| Thermoregulation | 1.65 ± 1.91 (n=407) | 1.97 ± 1.89 (n=108) | 1.53 ± 1.90 (n=299) | 0.038 |
| Fatigue A | 8.19 ± 2.46 (n=407) | 8.61 ± 2.48 (n=108) | 8.03 ± 2.43 (n=299) | 0.036 |
| Fatigue B | 16.81 ± 1.84 (n=407) | 16.19 ± 1.99 (n=108) | 17.03 ± 1.74 (n=299) | <0.001 |
| Sleep | 5.67 ± 3.32 (n=407) | 6.37 ± 3.78 (n=108) | 5.42 ± 3.10 (n=299) | 0.020 |
| Recovery | 5.94 ± 2.37 (n=407) | 6.45 ± 2.43 (n=108) | 5.76 ± 2.33 (n=299) | 0.009 |
| Energy/mood | 4.74 ± 2.51 (n=407) | 5.14 ± 2.62 (n=108) | 4.60 ± 2.46 (n=299) | 0.054 |
| Sex drive (items 1–2 only) | 0.53 ± 1.12 (n=407) | 0.48 ± 1.12 (n=108) | 0.54 ± 1.12 (n=299) | 0.631 |

Note. Values are mean ± SD with valid n shown in parentheses. Determined by independent-samples t tests.

**Supplementary Table S2. DASS-21 depression, anxiety, and stress symptom burden (N = 400)**

| **Scale** | **M (SD)** | **Moderate+ n (%)** | **Cronbach's α** |
| --- | --- | --- | --- |
| Depression | 7.69 (8.27) | 84 (21.0%) | .900 |
| Anxiety | 5.42 (5.90) | 85 (21.3%) | .751 |
| Stress | 11.31 (8.22) | 73 (18.3%) | .842 |

Note. DASS-21 subscale scores are on the 0–42 metric (items recoded 0–3; subscale sums multiplied by 2). Moderate+ thresholds: Depression ≥14, Anxiety ≥10, Stress ≥19.

**Supplementary Table S3. Interaction effects of psychosocial and climbing factors on the LEA–injury association**

| **Interaction model term** | **b (SE)** | **β (OR)** | **p** |
| --- | --- | --- | --- |
| Model fit (EDE-QS interactions) |  |  |  |
| LEAM (z) × EDE-QS (z) | 0.284 (0.140) | 1.328 | 0.042 |
| EDE-QS (z) × Female | 0.055 (0.248) | 1.057 | 0.823 |
| Model fit (Anxiety) |  |  |  |
| LEAM (z) × Anxiety (z) | 0.147 (0.123) | 1.158 | 0.232 |
| Model fit (Depression) |  |  |  |
| LEAM (z) × Depression (z) | 0.166 (0.123) | 1.180 | 0.177 |
| Model fit (Bouldering × ability) |  |  |  |
| LEA × Bouldering | -0.113 (0.260) | 0.893 | 0.663 |
| LEA × Higher ability | -0.366 (0.256) | 0.693 | 0.153 |
| Bouldering × Higher ability | 0.207 (0.557) | 1.230 | 0.710 |

Note. LEAM and EDE-QS exposures were standardized (z-scores) so ORs reflect odds per 1-SD increase. Interaction ORs reflect multiplicative change in the LEAM–injury association per 1-SD increase in the moderator, or the corresponding categorical contrast for climbing-context terms. Performance level is a dichotomous higher-ability indicator (ref=lower).

**Supplementary Table S4. One-domain-at-a-time revised LEAM domain models for current injury (each model adjusted; N=390)**

| **Domain** | **OR** | **95% CI** | **p** | **N** |
| --- | --- | --- | --- | --- |
| dizziness | 1.407 | 1.118, 1.771 | 0.004 | 390 |
| GI | 1.407 | 1.120, 1.768 | 0.003 | 390 |
| thermoregulation | 1.203 | 0.942, 1.536 | 0.138 | 390 |
| fatigue A | 1.327 | 1.032, 1.706 | 0.027 | 390 |
| fatigue B | 0.585 | 0.457, 0.748 | <0.001 | 390 |
| sleep | 1.297 | 1.030, 1.632 | 0.027 | 390 |
| recovery | 1.365 | 1.075, 1.733 | 0.011 | 390 |
| energy | 1.282 | 1.010, 1.627 | 0.041 | 390 |
| sex drive (items 1–2 only) | 0.929 | 0.732, 1.179 | 0.546 | 390 |

Note. Each model was adjusted for age, sex, climbing ability, training frequency, and climbing frequency. Domain predictors were standardized (z-scores).

**Supplementary Table S5. Full multivariable domain model (all LEAM domains entered together) for current injury (N=390)**

| **Predictor** | **OR** | **95% CI** | **p** |
| --- | --- | --- | --- |
| Z LEAM dizziness | 1.206 | 0.937, 1.554 | 0.146 |
| Z LEAM GI | 1.298 | 1.014, 1.661 | 0.038 |
| Z LEAM thermoregulation | 1.021 | 0.776, 1.342 | 0.883 |
| Z LEAM fatigue A | 1.013 | 0.735, 1.394 | 0.939 |
| Z LEAM fatigue B | 0.588 | 0.453, 0.764 | <0.001 |
| Z LEAM sleep | 1.098 | 0.827, 1.458 | 0.518 |
| Z LEAM recovery | 1.331 | 0.967, 1.831 | 0.079 |
| Z LEAM energy | 1.025 | 0.722, 1.456 | 0.889 |
| Z LEAM sex drive (items 1–2 only) | 0.845 | 0.647, 1.104 | 0.217 |
| Age_combined | 1.013 | 0.985, 1.042 | 0.355 |
| Female (ref=Male) | 0.919 | 0.515, 1.638 | 0.774 |
| Higher ability (ref=Lower) | 2.669 | 1.504, 4.736 | <0.001 |
| Training freq: Moderate (ref=Low) | 1.155 | 0.651, 2.049 | 0.622 |
| Training freq: High (ref=Low) | 1.485 | 0.717, 3.075 | 0.287 |
| Climbing freq: Moderate (ref=Low) | 0.528 | 0.280, 0.998 | 0.049 |
| Climbing freq: High (ref=Low) | 0.422 | 0.189, 0.941 | 0.035 |

Note. Domain coefficients represent unique associations after accounting for overlap across domains and covariates.

**Supplementary Table S6. Parsimonious multivariable domain model (selected LEAM domains) for current injury (N=390)**

| **Predictor** | **OR** | **95% CI** | **p** |
| --- | --- | --- | --- |
| Z LEAM sleep | 1.125 | 0.855, 1.480 | 0.400 |
| Z LEAM recovery | 1.211 | 0.893, 1.643 | 0.217 |
| Z LEAM energy | 0.952 | 0.684, 1.324 | 0.770 |
| Z LEAM GI | 1.337 | 1.054, 1.695 | 0.017 |
| Z LEAM thermoregulation | 1.098 | 0.849, 1.421 | 0.476 |
| Z LEAM fatigue A | 1.135 | 0.833, 1.547 | 0.423 |
| Age_combined | 1.012 | 0.985, 1.040 | 0.382 |
| Female (ref=Male) | 0.991 | 0.563, 1.747 | 0.976 |
| Higher ability (ref=Lower) | 2.277 | 1.313, 3.951 | 0.003 |
| Training freq: Moderate (ref=Low) | 1.160 | 0.668, 2.014 | 0.598 |
| Training freq: High (ref=Low) | 1.428 | 0.706, 2.887 | 0.322 |
| Climbing freq: Moderate (ref=Low) | 0.585 | 0.317, 1.081 | 0.087 |
| Climbing freq: High (ref=Low) | 0.485 | 0.225, 1.046 | 0.065 |

Note. Domain coefficients represent unique associations after accounting for overlap across retained domains and covariates.
